# Functional Profiling of the A-Family of Venom Peptides from the Wolf Spider *Lycosa shansia*

**DOI:** 10.3390/toxins15050303

**Published:** 2023-04-22

**Authors:** Tim Lüddecke, Ludwig Dersch, Lennart Schulte, Sabine Hurka, Anne Paas, Markus Oberpaul, Johanna Eichberg, Kornelia Hardes, Sven Klimpel, Andreas Vilcinskas

**Affiliations:** 1 Department of Bioresources, Fraunhofer Institute for Molecular Biology and Applied Ecology, Ohlebergsweg 12, 35392 Giessen, Germany; 2 LOEWE Centre for Translational Biodiversity Genomics (LOEWE-TBG), Senckenberganlage 25, 60325 Frankfurt, Germany; 3 Institute for Insect Biotechnology, Justus Liebig University Giessen, Heinrich-Buff-Ring 26-32, 35392 Giessen, Germany; 4 BMBF Junior Research Group in Infection Research “ASCRIBE”, Ohlebergsweg 12, 35392 Giessen, Germany; 5 Institute for Ecology, Evolution and Diversity, Goethe University Frankfurt, Max-von-Laue-Str. 13, 60439 Frankfurt am Main, Germany; 6 Senckenberg Gesellschaft für Naturforschung, Senckenberg Biodiversity and Climate Research Centre, Senckenberganlage 25, 60325 Frankfurt am Main, Germany

**Keywords:** spider venom, *Lycosa shansia*, venomics, antibiotics, drug discovery, insecticides, cytotoxicity, antiviral activity, influenza, in silico assessment

## Abstract

The venoms of spiders from the RTA (retro-lateral tibia apophysis) clade contain diverse short linear peptides (SLPs) that offer a rich source of therapeutic candidates. Many of these peptides have insecticidal, antimicrobial and/or cytolytic activities, but their biological functions are unclear. Here, we explore the bioactivity of all known members of the A-family of SLPs previously identified in the venom of the Chinese wolf spider (*Lycosa shansia*). Our broad approach included an in silico analysis of physicochemical properties and bioactivity profiling for cytotoxic, antiviral, insecticidal and antibacterial activities. We found that most members of the A-family can form α-helices and resemble the antibacterial peptides found in frog poison. The peptides we tested showed no cytotoxic, antiviral or insecticidal activities but were able to reduce the growth of bacteria, including clinically relevant strains of *Staphylococcus epidermidis* and *Listeria monocytogenes*. The absence of insecticidal activity may suggest that these peptides have no role in prey capture, but their antibacterial activity may help to defend the venom gland against infection.

## 1. Introduction

Spider venoms are complex mixtures of proteins, peptides and metabolites that are primarily used to overpower prey following delivery via a cheliceral injection system [1]. The venoms are predominantly neurotoxic, causing rapid paralysis and death [1,2,3]. The individual venom components have specific toxic effects but may also act synergistically to potentiate the neurochemical impact [1,4,5,6]. The main components of most spider venoms are small cysteine-rich peptides with a pseudoknot motif formed by disulfide bonds, often described as inhibitor cysteine knot (ICK) peptides [1,2,3]. Such peptides are potent modulators of ion channels and receptors and are primarily responsible for the neurotoxic effects of envenomation [1,2,3]. Several ICK peptides interact with pharmacologically relevant targets in humans and insects, and have therefore been investigated as drug candidates and bioinsecticides [2,7,8,9,10,11]. The functional properties of other polypeptide components in spider venom have yet to be evaluated [1].

**Figure 1:**
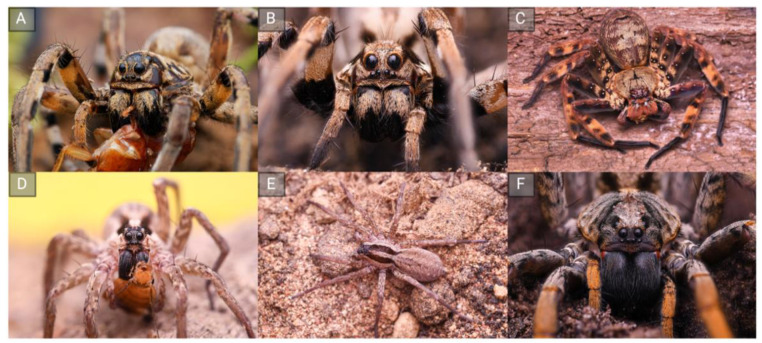
The diversity of RTA-clade spiders, the only spiders known to produce short linear peptides (SLPs) in their venoms. Top row, from left to right: *Lycosa hispanica* (**A**), *Lycosa praegrandis* (**B**) and *Heteropoda lunula* (**C**). Bottom row, from left to right: *Hogna graeca* proximal (**D**) plus dorsal (**E**) views, and *Hogna maderiana* (**F**). Image credit: Louis Roth, German Arachnological Society.

Recent technological developments in venom research have allowed the analysis of the greater diversity within the global arachnofauna [1,12,13,14,15,16]. The resulting data suggest the a priori assumption that ICK peptides, which dominate spider venoms, are not applicable to all spider taxa, particularly araneomorphs. A particular interesting example is the venoms of wandering spiders from the so-called RTA clade (Figure 1), a large monophyletic group including several spider families that is defined by the presence of a retro-lateral tibia apophysis (RTA) on the male pedipalps. This group has recruited another major peptide class known as that of short linear peptides (SLPs) into its venom [1]. SLPs are also described as cytolytic peptides, cationic peptides or spider venom antimicrobial peptides (AMPs) [17,18]. Interestingly, such peptides have been identified in RTA-clade venoms but not in any other spider venoms [18]. They range from 10 to 30 amino acids in length and several of them can be expressed on a single polycistronic transcript. Overall, they are cationic peptides that form amphipathic α-helices in the proximity of lipid bilayers [3]. They interact with a variety of biomembranes and form pores via different mechanisms, explaining their cytolytic activity [3]. Many of such peptides are active against bacteria, hence they can be affiliated with AMPs. However, several members of this class have acquired additional bioactivities, including cytotoxicity and neurotoxicity against insects [19,20]. At the time of writing, the biological function of SLPs remains unclear. Their antibacterial activity may defend the venom gland against microbial colonization. Their cytolytic activity may help to break down tissue at the injection site and foster the spread of co-injected neurotoxins (spreading factors) or support extra-intestinal digestion. However, because cytolysis causes pain, SLPs may also act as defensive weapons to deter predators. Finally, their neurotoxic effects in insects may play a role in trophic envenomation. These activities may be exploited for different aspects. The antimicrobial effects are especially promising because they could be used to develop novel antibiotics [21,22,23].

Despite their translational potential, little is known about SLPs and many of the identified SLPs in RTA-clade spiders are not yet functionally characterized. Even when bioactivity data are available, screenings usually focus on a few selected bioactivities, mostly cytotoxicity and/or antibacterial effects. Because SLPs can display multiple biological activities, broader screening pipelines are needed to fully grasp the functional space of each component, and by extension to determine their biological roles and translational potential.

One example of the diverse array of uncharacterized SLPs is the peptides from the venom of the Chinese wolf spider *Lycosa shansia*, previously known as *Lycosa sinensis* [24]. This spider was the recent target of mass spectrometry-based venom profiling, and a set of 52 SLPs was identified [24]. These were assigned to eight distinct families (A–H) by phylogenetic analysis [24]. The most diverse is the A-family, but bioactivity assays have yet to be reported [24]. Therefore, in this study we set out to investigate the function and potential applications of this important family of peptide toxins. We synthesized all known members and performed broad bioactivity profiling based on in silico analyses and assays for determining insecticidal, antibiotic, antiviral and cytotoxic effects.

## 2. Results

**Figure 2:**
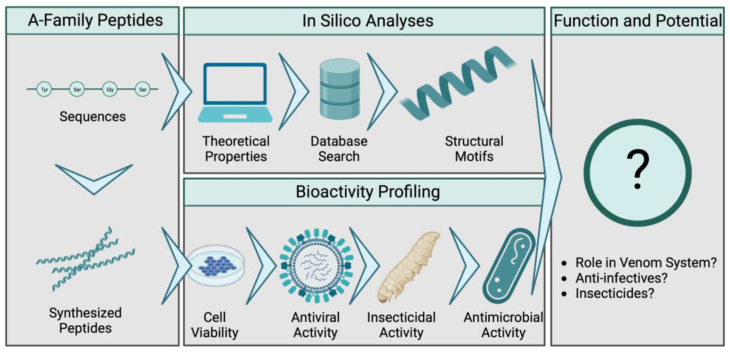
Workflow to determine the bioactivity of *L. shansia* A-family venom peptides. All known members of the family were synthesized and the physicochemical properties and structures were predicted in silico. Injection into *G. mellonella* larvae was performed to test for insecticidal activity. Cell viability assays were used to study cytotoxicity against MDCK II cells and protective effects against influenza (H1N1, H3N2 and B-Malaysia). Antibacterial assays were used to characterize antibiotic effects.

We applied a broad strategy for functional characterization that included in silico analysis combined with experimental bioassays (Figure 2). Firstly, all known A-family members were synthesized and their sequences were used to predict their physicochemical properties, secondary structures and closest relatives among the known AMPs. The synthetic peptides were then tested in our bioactivity profiling pipeline against a variety of target organisms. Cytotoxic activity was assessed in viability assays using mammalian cells (MDCK II and A549 cell lines). Insecticidal activity was tested by injection into the larvae of the greater wax moth, *Galleria mellonella*. Antiviral activity was tested against the influenza virus (A and B). Finally, we used a screening panel of seven bacterial strains to determine potential antibacterial activities.

### 2.1. Physicochemical Properties and Structures of A-Family Peptides

We determined the primary structure of the known *L. shansia* A-family venom peptides. The shortest was only 10 amino acids in length (LS-AMP-A20) whereas the longest was 20 amino acids (LS-AMP-A19) in length and the average length was 17. The hydrophobicity ranged from 30% (LS-AMP-A20) to 53% (LS-AMP-A3). Three peptides were predicted to be neutral (LS-AMP-A8, -A9 and -A11) whereas most were cationic, carrying either +2 charges (LS-AMP-A5, -A10 and -A20) or +1 charges (all the remaining peptides) (Table 1).

**Figure 3:**
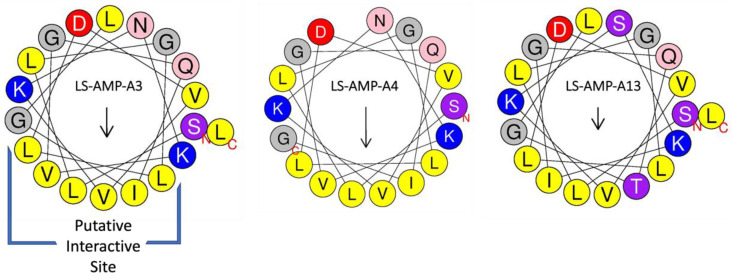
Structure of selected A-family peptides as determined by HeliQuest. Three representative members of the family are shown as helical wheel projections. Arrows indicate the hydrophobic moment, with longer arrows indicating greater relative strength. For helical wheel projections and values of hydrophobic moments, see Supplementary File S1. The putative interactive site is indicated in brackets for LS-AMP-A3.

The A-family peptides from *L. shansia* venom are predicted to form α-helices [24]. We investigated their ability to fold into helical structures in silico using HeliQuest software, and the results supported the α-helical structure of most of the peptides, with only LS-AMP-A20 as an obvious exception. This analysis also revealed that A-family peptides are amphipathic, featuring a hydrophobic face with a hydrophobic moment (Figure 3). This structure, possibly representing the interaction site of A-family peptides, contains a characteristic LTVLVL motif that is present in most members. However, some of the peptides include amino acid replacements or deletions in the hydrophobic face, such as the replacement of tryptophan with isoleucine (LTVL to LIVL) in LS-AMP-A3, -A4 and -A5. The structures with calculated hydrophobic moments and hydrophobic faces are summarized in Supplementary File S1.

### 2.2. A-Family Peptides Resemble AMPs from Frog Poisons

To determine whether the A-family peptides are similar to known AMPs, we used them as queries to screen the AMP database (APD3). Interestingly, the A-family peptides and AMPs previously isolated from the skin poison of tree frogs in the genus *Litoria* (Pelodryadinae) share ~50% sequence similarity (Table 2). Most of the A-family peptides were most closely related to *L. aurea* Aurein 2.1, but two each showed greater similarity to *L. splendida* Caerin 2.1 and *L. eucnemis* Maculatin 1.3, and one each showed greater similarity to *L. caerula* Caerin 2.6 and *L. aurea* Aurein 2.4.

### 2.3. Evaluation of Cytotoxicity

**Figure 4:**
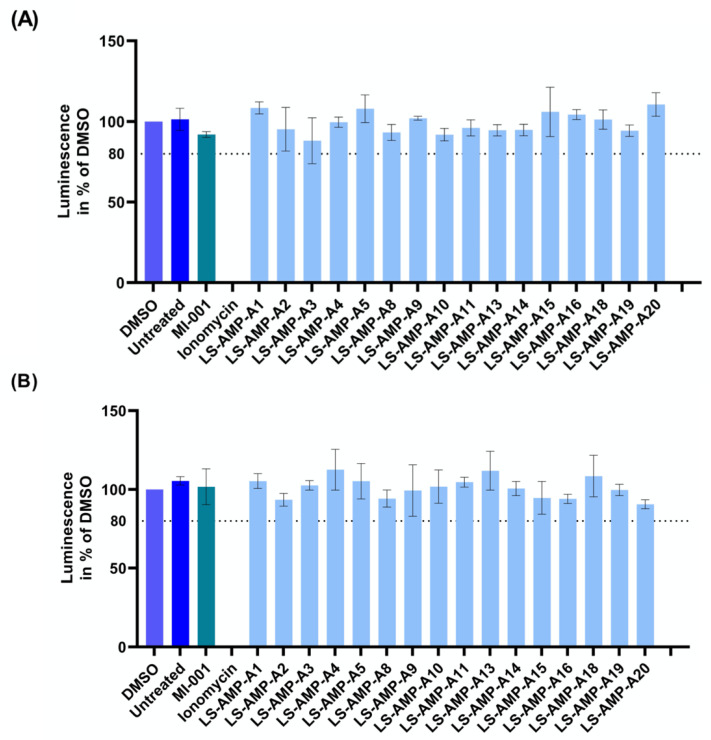
Effect of A-family peptides on cell viability. (**A**) Viability of MDCK II cells. (**B**) Viability of A549 cells. The cells were treated with a 100 µM concentration of each peptide or DMSO as a solvent control. Cell viability was assessed 48 h after treatment via CellTiterGlo Assay. The viability of DMSO-treated cells was set at 100%. Results are mean values ± SD (*n* = 3).

AMPs primarily interact with bacterial membranes, creating pores that cause cytoplasmic leakage [3]. However, they can also show toxicity toward eukaryotic cells [3]. We therefore investigated their effect against mammalian cells. Specifically, we examined the effects on cell viability of two cell lines (MDCK II and A549). We found that that none of the A-family peptides had any effect on cell viability, even at the high concentration of 100 μM (Figure 4). Raw data are given in Supplementary File S2.

### 2.4. Evaluation of Antiviral Activity

**Figure 5:**
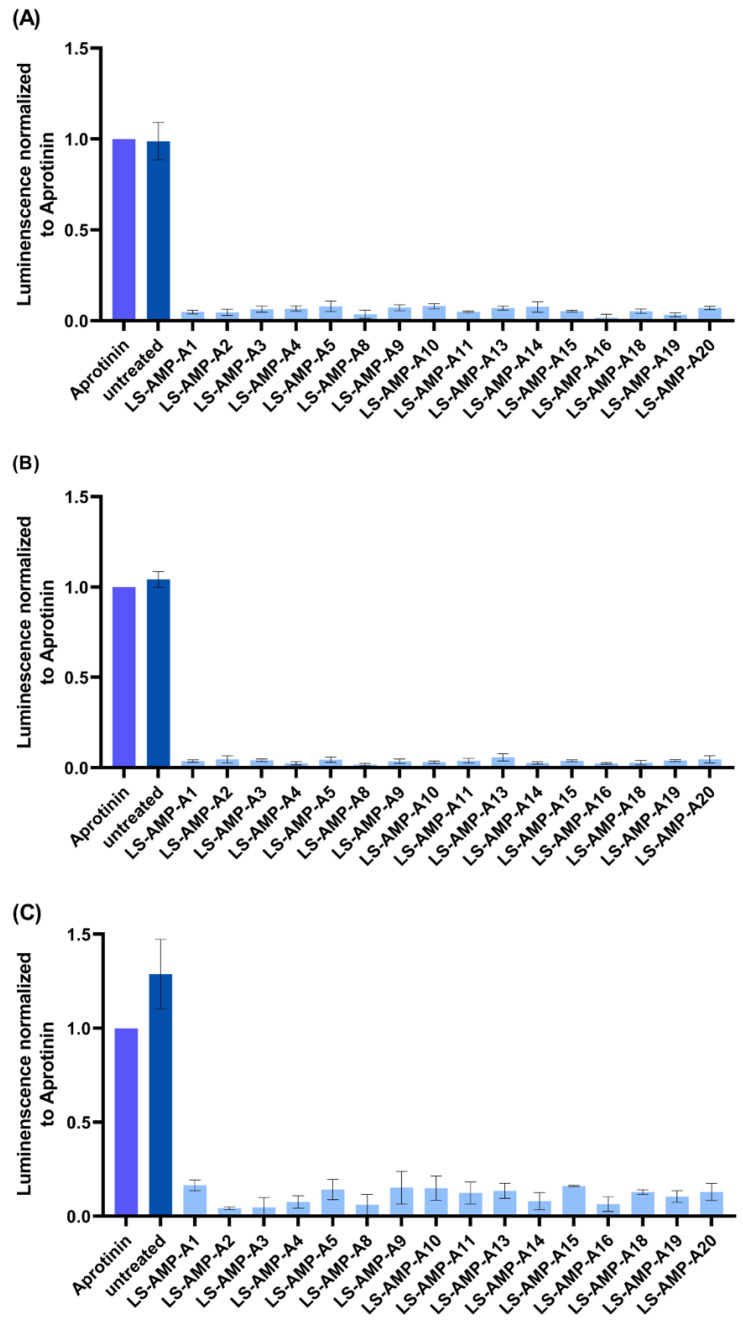
Antiviral effects of A-family peptides against influenza B-Mal (**A**), H1N1 (**B**) and H3N2 (**C**). Near-confluent MDCK II cells were infected with a virus at MOI = 1. Infected cells were treated with selected peptides at a concentration of 100 µM or left untreated as a cytopathic control. After incubation at 37 °C for 48 h, cell viability was determined by CellTiterGlo Assay. The viability of aprotinin-protected cells was set to one. Results are mean values ± SD (*n* = 3).

Some linear venom peptides are capable of exerting antiviral activity, including activities against influenza viruses [25,26,27]. Given that the A-family peptides showed no toxicity toward MDCK II cells, we infected these cells with influenza viruses (H1N1, H3N2 or B-Malaysia (B-Mal)) and determined whether or not the A-family peptides possess antiviral activity. We assessed the virus-induced cytopathic effect in cells treated with the peptides compared to aprotinin-protected cells as a control. We found no evidence of antiviral activity (Figure 5). Raw data are given in Supplementary File S3.

### 2.5. Insecticidal Activity Evaluation against G. mellonella Larvae

**Figure 6:**
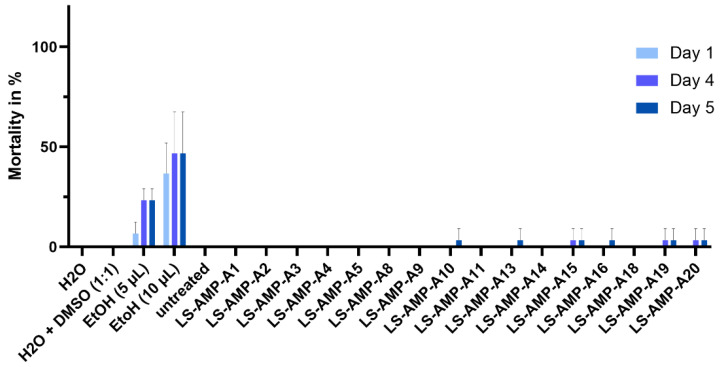
Insecticidal assay using A-family peptides injected in *G. mellonella* larvae and monitoring of their survival for 5 d. Larvae were injected with water, 50:50 water: DMSO (*v*/*v*) or ethanol as controls. None of the peptides showed insecticidal activity.

We injected each of the A-family peptides into *G. mellonella* larvae and monitored their survival over a period of 5 days (Figure 6). Ethanol was injected as a positive control, resulting in up to 50% mortality after 5 days, whereas water and 50:50 water:DMSO (*v*/*v*) had no effect. Similarly, none of the peptides had an effect on the larvae. Individual dead insects were found among the cohorts injected with LS-AMP-A10, -A13, -A15, -A16, -A16, -A19 and -A20. Raw data are given in Supplementary File S4.

### 2.6. Antibacterial Activity

**Figure 7:**
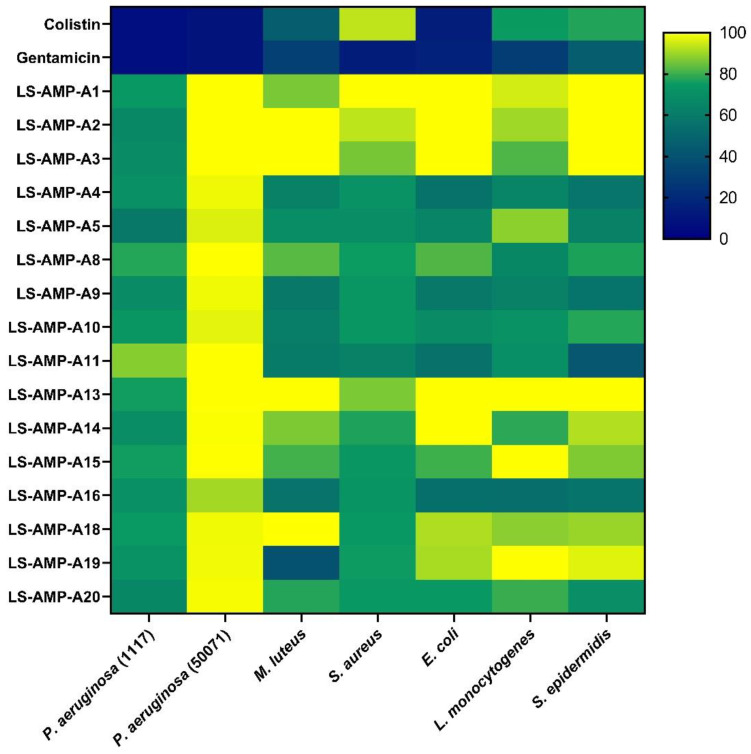
Activity of A-family peptides against seven bacterial strains. The heat map is based on bacterial growth measured by spectrophotometry after peptide exposure for 48 h. Growth rates were calculated in relation to growth on unmodified media. Gentamicin and colistin were used as a control. Brighter colors indicate higher growth rates and darker colors indicate growth inhibition.

Finally, we assessed the ability of the synthetic A-family peptides to inhibit the growth of bacteria, including several clinically relevant strains (Figure 7). LS-AMP-A1, -A2 and -A3 showed limited activity. LS-AMP-A1 inhibited the growth of *Pseudomonas aeruginosa* DSM 1117 and *Listeria monocytogenes* DSM 20600 by ~50% after 48 h, whereas LS-AMP-A2 and -A3 were less effective. In contrast, LS-AMP-A4, -A5, -A8, -A9, -A10 and -A11 inhibited the growth of most of the bacteria except two: the strains *P. aeruginosa* DSM 1117 and *P. aeruginosa* DSM 50071. LS-AMP-A5, -A8 and -A10 also showed no activity against *Micrococcus luteus* DSM 20030. LS-AMP-A13 and -A15 showed limited activity against *Staphylococcus aureus* DSM 2569 and *S. epidermidis* DSM 28319, but none of the other bacteria. LS-AMP-A14 showed weak activity against *P. aeruginosa* DSM 1117 and *P. aeruginosa* DSM 50071 but was more active against *L. monocytogenes* DSM 20600. LS-AMP-A16 showed broad activity against all tested bacteria except *P. aeruginosa* DSM50071. LS-AMP-A18 and LS-AMP-A19 were largely inactive, although the latter strongly suppressed the growth of *M. luteus* DSM 20030. Finally, LS-AMP-A20 showed marginal effects against several bacteria, but the strongest effect was observed against *M. luteus* DSM 20030. The raw data for each peptide and bacterium are presented in Supplementary File S5.

## 3. Discussion

Spider venoms contain a vast repertoire of bioactive peptides with potential applications in medicine and agriculture, but most of such molecules have yet to be characterized [2,9]. One good example is the class of short linear peptides (SLPs) found in the venoms of araneomorph spiders representing the RTA clade [1]. We performed an in silico analysis along with a broad range of bioactivity assays to discover the functions of an entire SLP family from wolf spiders.

Venoms are used mainly for predation, defense and intraspecific interactions [28]. In spiders, the first two purposes are predominant and most spider venom components therefore act on targets in arthropods (prey) and vertebrates (defense) [1]. The biological functions of the better-studied components, such as ICK peptides, have been investigated in detail, but much less is known about SLPs. Most of the SLPs investigated thus far have shown antibacterial effects, but some are cytotoxic and even insecticidal [19,29,30,31]. SLPs with the same molecular function may have different biological purposes. For example, insecticidal compounds probably function in a trophic scenario but could also defend the host against threats from larger arthropods. Cytotoxic SLPs could act as spreading factors to facilitate the uptake of co-injected neurotoxins by damaging the cells at the injection site [6]. However, cell death is also likely to cause painful local symptoms and could therefore be used to produce painful defensive bites. In combination with the threat postures often displayed by RTA-clade spiders, such painful effects could help to trigger learning behavior in intelligent vertebrate predators, ultimately leading to the avoidance of similar-looking prey [1]. The breakdown of tissue after envenomation could support the extra-intestinal digestion performed by spiders, suggesting that SLPs have a pseudo-trophic function. Finally, antibacterial activity could help to defend the venom gland against microbial infection, therefore contributing to the immunity of the venom system. SLPs may even fulfil multiple functions that differ among the distinct SLP families.

The A-family of SLPs was recently identified in *L. shansia* venom but their functions have not been characterized [24]. We found that A-family peptides show no toxicity toward mammalian MDCK II and A549 cells (Figure 4), suggesting that they are unlikely to cause pain to envenomated mammals. Accordingly, they are unlikely to function in a defensive capacity, and are probably not spreading factors or pseudo-trophic components that support extra-intestinal digestion. We also found no evidence of significant insecticidal activity when the A-family peptides were injected into *G. mellonella* larvae (Figure 6). Seven of the peptides killed individual larvae within 48 h but this weak effect is not indicative of an insecticidal function. In trophic scenarios, lethal and/or paralyzing activity must stimulated immediately after envenomation to ensure that prey cannot escape. A-family peptides are therefore unlikely to be used for prey capture. 

Finally, we determined whether A-family peptides were able to inhibit pathogens. The peptides showed negligible protective effects against influenza viruses, indicating the absence of antiviral activity (Figure 5). However, several of the peptides showed relatively broad but not tremendously potent activity against a panel of bacteria. LS-AMP-A4, -A9, -A11 and -A16 were able to decrease the growth of *M. luteus* DSM 20030, *L. monocytogenes* DSM 20600, *S. aureus* DSM 2569, *S. epidermidis* DSM 28319 and *E. coli* BL21(DE3) (Figure 7). The panel of bacteria included several clinically relevant strains, including the food-borne pathogen *L. monocytogenes* DSM 20600, which causes listeriosis and meningitis in newborns [32,33,34]. However, even the best-performing A-family peptides showed limited potency, typically inhibiting bacterial growth by less than 50%. The native, sole peptides are therefore unattractive for development as novel antibiotics despite their weak cytotoxicity. Regardless, herein, we only investigated singular toxins and omitted an analysis that investigates the potential synergistic actions between peptides. Thus, testing a mixture of peptides and also performing structural modifications based on the native sequences may offer a more suitable set of candidates. Accordingly, future studies should investigate the synergistic activities of these peptides in more detail.

Interestingly, A-family peptides are similar to several AMPs isolated from the skin poison of *Litoria* tree frogs (Table 2) [35,36,37,38,39,40]. In amphibian skin poison, AMPs are important components that apply their broad-spectrum antibacterial activity to defend unprotected skin against pathogens [41]. The natural function of A-family peptides and *Litoria* AMPs may therefore be similar. AMPs work by interacting with membranes, penetrating the lipid bilayer and forming pores via different mechanisms [3]. The physicochemical properties of the cell surface and peptide determine the nature of the interaction, and the hydrophobic site, especially, is a key mediator in this relationship [3]. In the A-family peptides, we identified a putative interaction site containing an LTVLVL motif that forms a hydrophobic face. Interestingly, *Litoria* AMPs display similar hydrophobic sites, which could explain their functional alignment with A-family SLPs (Supplementary File S1).

Given the structural and functional similarities between A-family peptides and *Litoria* AMPs, and taking the general biological role of amphibian AMPs into account, we hypothesize that the principal biological role of *L. shansia* A-family peptides is to reduce growth rates of bacteria entering the venom system and thus help to prevent potential infections. The lumen of the venom gland is a large body cavity filled with proteins and nutrients. Uncontrolled microbial infection in such an environment could lead to rapid replication and potentially lethal infections. The gland is connected to the chelicerae by a venom duct [42,43]. The envenomation of targets (attackers or prey) therefore provides an opportunity for inoculation with microbes that could develop into an infection. In this context, antibacterial components such as those of the A-family peptides may act as extended branches of the spider immune system inside the venom gland. Although venom has traditionally been considered a sterile mixture, recent studies have shown that spider venoms are reservoirs for different microbial taxa, including some pathogens [44,45]. Therefore, the antimicrobial peptides in RTA-clade venom systems may also help to maintain a stable microbiome that defends the venom gland against infection. 

## 4. Conclusions

The venoms of spiders in the RTA clade uniquely contain SLPs. Many of these have not been characterized in detail so we know little about their biological functions or translational potential. Our work has shed light on the functions of one SLP family (the A-family) from the Chinese wolf spider *L. shansia*. The A-family peptides were observed to share physicochemical properties with AMP-like toxins previously isolated from from *Litoria* tree frog poison. Our bioactivity screenings revealed that the A-family peptides are neither insecticidal nor cytotoxic and are unlikely to be involved in trophic envenomation. They also have no significant antiviral activity, but most of the peptides were able to inhibit the growth of bacteria, including clinically relevant *L. monocytogenes* and *S. epidermidis* strains. The native peptides are not potent enough for drug development, although derivatives with optimized activity may be suitable candidates. Based on their similarity to amphibian skin poison compounds with antibacterial activity, we hypothesize that A-family peptides work as broad-spectrum antibiotics that help to defend the venom gland from microbial colonization and infection. Future studies should investigate more families of spider SLPs and screen more spider venoms to understand their distribution and function across different taxa.

## 5. Materials and Methods

### 5.1. Sequence Analysis

The sequences of A-family peptides were retrieved from the original study [24]. Multiple sequence alignments were produced using MUSCLE in Geneious (https://www.geneious.com). The AMP calculator and predictor tool of the AMP database v3 (APD3) [46] and the HeliQuest analysis tool [47] were used to determine the physicochemical properties of all peptides. The APD3 predictor tool was also used to identify known AMPs with the greatest similarity to the A-family. The HeliQuest analysis tool was used to produce helical wheel projections of the A-family peptides using default settings for α-helices under the full sequence criterion.

### 5.2. Peptide Synthesis

Peptide synthesis was outsourced to a commercial supplier of synthetic biomolecules (GenScript Biotech, Rijswijk, The Netherlands) via their AccuPep technology [48,49,50]. An amount of 10 mg of each peptide was produced in 1 mg aliquots by solid-phase synthesis followed by lyophilization. Quality control was performed by the supplier via HPLC and mass spectrometry to validate the molecular weight and determine the purity of each component (Table 3). Peptides known to carry a C-terminal amide were synthesized in amidated form.

### 5.3. Cell Culture

Madin-Darby canine kidney II (MDCK II) cells were used to investigate cytotoxic and antiviral effects. Cells were maintained in Dulbecco’s modified Eagle’s medium (DMEM GlutaMAX) supplemented with 1% penicillin/streptomycin and 10% fetal calf serum (all reagents were obtained from Thermo Fisher Scientific, Walthman, MA, USA). They were grown in an incubator at 37 °C in a 5% CO_2_ atmosphere.

### 5.4. Cytotoxicity Assays

Cytotoxicity was determined as previously described [31]. Peptides and ionomycin (Cayman Chemical, Ann Arbor, MI, USA) were dissolved in DMSO to create 10 mM stock solutions. MDCK II (kidney epithelial cells) and A549 (epithelial lung carcinoma cells) cells were seeded and cultured to 90% confluence in 96-well plates and treated with the compounds (at 100 µM) or DMSO for 48 h at 37 °C in a 5% CO_2_ atmosphere. Cell viability was determined using CellTiterGlo Luminescent Cell Viability Assay (Promega, Walldorf, Germany). Luminescence was measured in black 96-well plates in a Synergy H4 microplate reader (Biotek, Waldbronn, Germany). Relative light units (RLU) were normalized to the DMSO control set at 100%. Triplicate measurements were used to calculate the means and standard deviations. Raw data are provided in Supplementary File S2.

### 5.5. Antiviral Activity

Antiviral activity was tested against three strains of influenza virus: A/Hamburg/05/2009 (H1N1), A/Hessen/1/2003 (H3N2) and B/Malaysia/2506/2004 (B-Mal). The serine protease inhibitor aprotinin (Carl Roth, Karlsruhe, Germany) and peptides were dissolved in water and stock solutions at a concentration of 10 mM were stored at −20 °C. Viruses were propagated in MDCK II cells in DMEM GlutaMAX supplemented with 1% penicillin/streptomycin, 0.2% bovine serum albumin (BSA, Thermo Fisher Scientific) and 1 µg/mL of TPCK-treated trypsin (Thermo Fisher Scientific). For antiviral screening, MDCK II cells were seeded to 90% confluence in an infection medium (as above, without trypsin) and inoculated with each virus strain at a multiplicity of infection of 1 (MOI = 1). After 1 h, the cells were washed twice with PBS, followed by treatment with each peptide at a 100 µM concentration or aprotinin in the full medium (including trypsin). Cell viability was determined 48 h after treatment using the CellTiterGlo assay, as described above. Virus-treated cells were used as blanks and RLU values were subtracted from the blank before normalizing them to aprotinin, set to one. Triplicate measurements were used to calculate means and standard deviations. Raw data are provided in Supplementary File S3.

### 5.6. Insecticidal Activity 

Insecticidal activity was determined using fourth and fifth instar *G. mellonella* larvae (L4/L5) obtained from Fauna Topics Zoobedarf Zucht und Handels GmbH (Marbach am Neckar, Germany). Because spider venom is injected via chelicerae, we opted for an injection-based assay to reflect the natural process of envenomation as described earlier [51]. The peptides were dissolved in a 1:1 mixture of DMSO and water to a final concentration of 10 mmol/L. We injected 50 ng of each peptide into the pseudopodium using Omnican-F, 0.30 × 12 mm/G 30 × 1/2”, a 1 mL syringe adjusted to a WPI manual microsyringe injector setup. Injections with water, DMSO/water (1:1) and ethanol (90%) were used as controls, alongside untreated specimens. Insecticidal activity screenings were performed in cohorts of 10 larvae with 3 replicates (30 specimens per treatment or control). Larvae were observed for 15 min after injection to detect any immediate effects. Survival was monitored every 24 h for 5 days. Mortality was calculated based on the ratio of dead to injected specimens. Raw data are given in Supplementary File S4.

### 5.7. Antibacterial Activity

Cryo-conserved cultures of selected bacterial strains (Table 4) were transferred to Tryptic Soy Agar (TSA) plates (Carl Roth) with a sterile inoculation loop, sealed with Parafilm (BEMIS, Neenah, WI, USA), and incubated for 1–2 days at 37 °C depending on growth. Single colonies were picked and transferred into 2–3 mL of the Mueller–Hinton II (MH II) broth (BD, Heidelberg, Germany). The bacteria were cultivated overnight, subcultured on fresh medium and grown for 3–4 h before testing at 37 °C, with shaking at 180 rpm. We measured the optical density at 600 nm (OD_600_) of 1 mL of the bacterial suspensions in polystyrene cuvettes (SARSTED, Nümbrecht, Germany) using an Ultrospec 10 spectrophotometer (Biochrome, Cambridge, UK). We then diluted them with the MH II medium to the preferred OD_600_ (Table 2). The peptides were dissolved in DMSO to generate 10 mmol/L stocks and diluted with the MH II medium to 200 µM before the assay. We used 10 µg/mL (~40 µg/mL for *S. epidermidis*) gentamicin and colistin (Sigma-Aldrich, Taufkirchen, Germany), DMSO and the medium only as controls. The bacteria were transferred to 96-multiwell plates with a final volume of 100 µL per well (50 µL bacterial suspension, and 50 µL test peptide). The OD_600_ was measured at 0, 24 and 48 h using a BioTek Eon microplate reader followed by analysis with Gen5 v2.09. Raw data are given in Supplementary File S5.

## Figures and Tables

**Table 1:** Properties of *L. shansia* A-family venom peptides. The table lists peptide names and sequences, as well as the number of amino acids (#AAs) and the predicted hydrophobicity and charge of each sequence. Peptides indicated with an asterisk are C-terminally amidated and thus carry higher charges when modified.

Peptide	Sequence	#AAs	Hydrophobicity	Charge
LS-AMP-A1	SLFGLLDLVKSKVGQTGLL	19	47%	+1
LS-AMP-A2	SLFGLLDLVKNKVGQTGLL	19	47%	+1
LS-AMP-A3	SLLGLLDVVKNKVGQIGLL	19	53%	+1
LS-AMP-A4	SLLGLLDVVKNKVGQIG	17	47%	+1
LS-AMP-A5 *	SLLGLLDVVKNKVGQI	16	44%	+2
LS-AMP-A8	SLLGLLDVVKNTVGQTGLL	19	47%	0
LS-AMP-A9	SLLGLLDVVKNTVGQTG	17	41%	0
LS-AMP-A10 *	SLLGLLDVVKNTVGQT	16	50%	+2
LS-AMP-A11	SLLGLLDVVKNTVGQ	15	47%	0
LS-AMP-A13	SLLGLLDVVKSKIGQTGLL	19	47%	+1
LS-AMP-A14	SLLGLLDVVKSKIGQTG	17	41%	+1
LS-AMP-A15 *	SLLGLLDVVKSKIGQT	16	44%	+1
LS-AMP-A16	LLDVVKSKIGQTGLL	15	47%	+1
LS-AMP-A18	SLLGLLDVVKSKVGQTGLL	19	47%	+1
LS-AMP-A19	GSLLGLLDVVKSKVGQTGLL	20	45%	+1
LS-AMP-A20 *	DVVKSKVGQT	10	30%	+2

* Mature peptides are amidated, as previously reported [24].

**Table 2:** Identification of known AMPs with the highest similarity to A-family peptides. The table lists the most similar hit for each A-family peptide, with percentage sequence similarity and source organism. Overall, A-family peptides resemble AMPs identified in skin poisons from tree frogs, frogs of the genus *Litoria*.

Name	Hit	Similarity	Species
LS-AMP-A1	Aurein 2.1	55%	*Litoria aurea*
LS-AMP-A2	Aurein 2.1	55%	*Litoria aurea*
LS-AMP-A3	Aurein 2.1	55%	*Litoria aurea*
LS-AMP-A4	Aurein 2.1	50%	*Litoria aurea*
LS-AMP-A5	Caerin 2.1	46%	*Litoria splendida*
LS-AMP-A8	Aurein 2.1	53%	*Litoria aurea*
LS-AMP-A9	Aurein 2.1	47%	*Litoria aurea*
LS-AMP-A10	Maculatin 1.3	43%	*Litoria eucnemis*
LS-AMP-A11	Maculatin 1.3	43%	*Litoria eucnemis*
LS-AMP-A13	Aurein 2.1	50%	*Litoria aurea*
LS-AMP-A14	Caerin 2.1	50%	*Litoria splendida*
LS-AMP-A15	Caerin 2.6	50%	*Litoria caerulea*
LS-AMP-A16	Aurein 2.1	53%	*Litoria aurea*
LS-AMP-A18	Aurein 2.1	55%	*Litoria aurea*
LS-AMP-A19	Aurein 2.1	52%	*Litoria aurea*
LS-AMP-A20	Aurein 2.4	39%	*Litoria aurea*

**Table 3:** Properties of produced peptides. Those shown were calculated via the mass spectrometry-determined molecular weights and the purity of each synthesized compound.

Peptide	MW calc.	MW. det.	Purity
LS-AMP-A1	1988.38	1989.2	82.0%
LS-AMP-A2	2015.41	2015.1	93.5%
LS-AMP-A3	1979.42	1979.2	96.8%
LS-AMP-A4	1753.10	1753.0	97.3%
LS-AMP-A5	1695.06	1695.2	87.8%
LS-AMP-A8	1940.29	1940.0	70.5%
LS-AMP-A9	1713.98	1714.0	82.4%
LS-AMP-A10	1655.94	1656.0	95.7%
LS-AMP-A11	1555.82	1555.8	93.4%
LS-AMP-A13	1954.36	1954.2	83.9%
LS-AMP-A14	1728.05	1727.8	90.6%
LS-AMP-A15	1670.01	1670.0	80.3%
LS-AMP-A16	1583.92	1583.8	92.0%
LS-AMP-A18	1940.34	1940.2	95.4%
LS-AMP-A19	1997.39	1997.1	89.4%
LS-AMP-A20	1059.22	1059.6	98.6%

**Table 4:** Taxonomic names, unique identifiers and the optimal OD_600_ assay values for our panel of test bacteria.

Name	Unique Identifier	OD_600_ for Assay
*Listeria monocytogenes*	DSM 20600	0.000625
*Micrococcus luteus*	DSM 20030	0.005
*Pseudomonas aeruginosa 50071*	DSM 50071	0.00125
*Pseudomonas aeruginosa 1117*	DSM 1117	0.005
*Staphylococcus aureus*	DSM 2569	0.00125
*Staphylococcus epidermidis*	ATCC 35984; DSM 28319	0.000625
*Escherichia coli DE3*	BL21(DE3)	0.000325

## Data Availability

Raw data obtained in this study are presented in the Supplementary Files.

## Supplementary Materials

The following supporting information can be downloaded at: https://www.mdpi.com/article/10.3390/toxins15050303/s1. Supplementary File S1: Overview of peptide structures and hydrophobic faces as predicted by HeliQuest. Supplementary File S2: Raw data from cytotoxicity assays against MDCK II cells. Supplementary File S3: Raw data of antiviral screenings against different influenza strains. Supplementary File S4: Raw data of insecticidal activity experiments. Supplementary File S5: Raw data of antimicrobial assays.
