# Supplementary material for: Functional Profiling of the A-Family of Venom Peptides from the Wolf Spider Lycosa shansia
Source: Toxins (Basel). 2023 Apr 22;15(5):303. doi: 10.3390/toxins15050303 (PMC10221074; doi:10.3390/toxins15050303)
Supplement: Supplementary file 1 [file toxins-15-00303-s001.zip › Supplementary File S1.pdf]

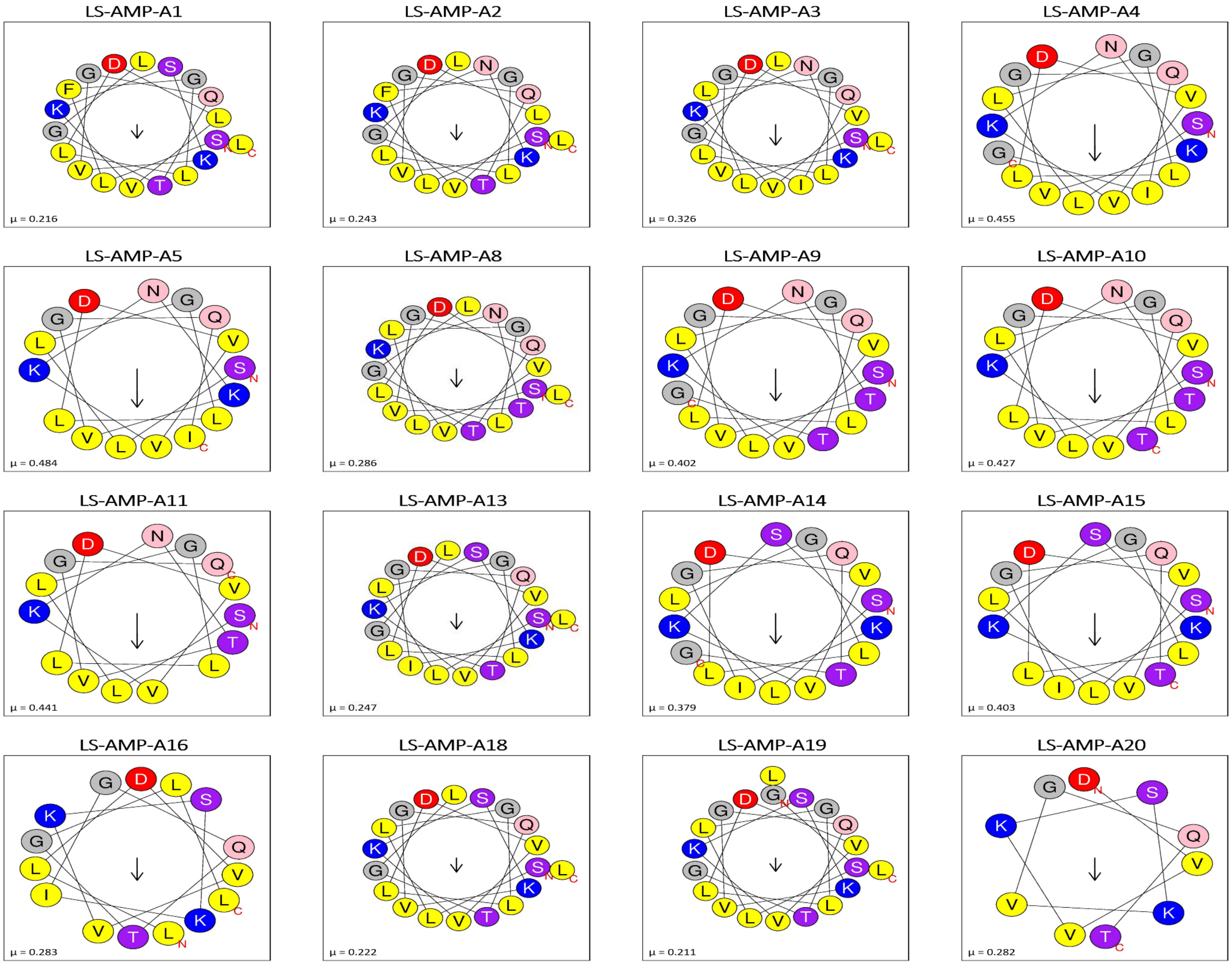
LS-AMP-A1
μ = 0.216
LS-AMP-A2
μ = 0.243
LS-AMP-A3
μ = 0.326
LS-AMP-A4
μ = 0.455
LS-AMP-A5
μ = 0.484
LS-AMP-A8
μ = 0.286
LS-AMP-A9
μ = 0.402
LS-AMP-A10
μ = 0.427
LS-AMP-A11
μ = 0.441
LS-AMP-A13
μ = 0.247
LS-AMP-A14
μ = 0.379
LS-AMP-A15
μ = 0.403
LS-AMP-A16
μ = 0.283
LS-AMP-A18
μ = 0.222
LS-AMP-A19
μ = 0.211
LS-AMP-A20
μ = 0.282
